# Tracking Multiple Marine Ships via Multiple Sensors with Unknown Backgrounds

**DOI:** 10.3390/s19225025

**Published:** 2019-11-18

**Authors:** Cong-Thanh Do, Tran Thien Dat Nguyen, Weifeng Liu

**Affiliations:** 1 School of Electrical Engineering, Computing, and Mathematical Sciences, Curtin University, Bentley, WA 6102, Australia; t.nguyen172@postgrad.curtin.edu.au; 2 School of Automation, Hangzhou Dianzi University, Hangzhou 310018, China; dashan_liu@163.com

**Keywords:** random finite sets, unknown background, bootstrapping method, GLMB filter, multisensor multitarget tracking, Murty’s algorithm

## Abstract

In multitarget tracking, knowledge of the backgrounds plays a crucial role in the accuracy of the tracker. Clutter and detection probability are the two essential background parameters which are usually assumed to be known constants although they are, in fact, unknown and time varying. Incorrect values of these parameters lead to a degraded or biased performance of the tracking algorithms. This paper proposes a method for online tracking multiple targets using multiple sensors which jointly adapts to the unknown clutter rate and the probability of detection. An effective filter is developed from parallel estimation of these parameters and then feeding them into the state-of-the-art generalized labeled multi-Bernoulli filter. Provided that the fluctuation of these unknown backgrounds is slowly-varying in comparison to the rate of measurement-update data, the validity of the proposed method is demonstrated via numerical study using multistatic Doppler data.

## 1. Introduction

In a multitarget scenario, the targets set cardinality and their dynamic states randomly vary with time. The objective of tracking multiple targets is to estimate the number of targets and their trajectories using the data collected from sensor(s) in a joint manner [1,2,3,4]. Currently, there are three major paradigms for this field of study, namely Joint Probability Data Association (JPDA) [1], Multiple Hypotheses Tracking (MHT) [2] and Random Finite Set (RFS) [3,4]. While the first two formers involve modifying single target tracking filters to accommodate the problem of multitarget tracking, the latter applies estimation theory focusing on Bayesian optimality and provide a top-down formulation for solving the multitarget estimation problem [3,4].

Using RFS leads to the development of a series of multitarget estimation algorithms. Several RFS-based filters has been proposed in both the literature and practical applications, such as the Probability Hypothesis Density (PHD) [5], Cardinalized PHD (CPHD) [6,7], and the multi-Bernoulli filters [8]. While these filters and their extensions can give good estimates of the current target states, they do not produce target trajectories without using heuristics [9,10]. A theoretically rigorous and systematic consideration of the multitarget trajectory estimation based on RFS approach was proposed in [11]. This work also derives an exact closed-form solution to the multitarget tracking problem, known as Generalized Labeled multi-Bernoulli (GLMB) filter. This filter can estimate not only the number of the targets but also their trajectories, simultaneously [12]. It has been applied to several problems as tracking with merged measurements [13], track-before-detect [14,15], extended targets [16], cell biology [17,18], sensor scheduling [19], spawning targets [20], distributed data fusion [21], field robotics [22,23] and computer vision [24]. The GLMB filter for multitarget tracking with two sensors has been developed in [25,26]. An efficient implementation of the GLMB filter based on Gibbs sampling whose complexity depends linearly on the total number of measurements and quadratically on the number of hypothesized targets has been presented in [27]. This method has been extended to the multi-scan GLMB filter [28] and the multi-sensor GLMB filter [9].

In the multitarget tracking problem, clutter and detection profile are notable sources of uncertainty [29]. Clutter is the set of false measurements that do not originate from any true target and detection profile models the ability of the sensor to detect targets. Knowledge of these parameters are essential in Bayesian multitarget estimation. Mismatches in parameters of clutter and detection models lead to poor performance of filtering algorithms. While these parameters are unknown and randomly time-varying, they are normally assumed to be known in advance. This assumption is unrealistic in most practical applications and these parameters need to be estimated from training data or manually tuned [29].

Since the adaptability of the tracker to these unknown parameters are important in practice, several RFS filters have been proposed in the literature to perform multitarget tracking with mismatches in clutter and detection probability. Some of the proposed methods that accommodate the unknown clutter rate are given in [30,31,32,33]. A filter which bootstraps the clutter estimator of [29] into the CPHD filter [6] has been proposed in [34]. Several approaches for dealing with unknown detection probability have been presented in the literature, such as [29,35,36]. However, none of these filters can output target tracks. While the GLMB filter can output tracks, and has been applied to several problems without prior knowledge of clutter rate, as in [37,38,39], it is still computationally expensive. A low computational cost bootstrapping method using GLMB filter has been given in [40] for multisensor multitarget tracking with unknown detection probability.

Multisensor multitarget tracking with jointly unknown clutter rate and detection profile is far more complicated than those with a single unknown parameter. The use of multiple sensors leads to multidimensional ranked assignment problem which is the main hurdle in the implementation of the GLMB filter [9]. Furthermore, exploiting background information from training data for the multitarget estimation at each time frame is insufficient due to the time-varying nature of the two mentioned unknown parameters.

This work is aimed to contribute an efficient method for multitarget tracking that not only produces target trajectories but also estimates the jointly unknown clutter rate and detection profile online with low computational cost. By using a simple combination of the two well-known filters, the CPHD and GLMB filters, this method is not only fast in estimating the unknown parameters but also producing trajectories of the targets. Specifically, these two mentioned unknown parameters would be estimated separately by using the λ-CPHD and $p_{D}-$CPHD filters before feeding to the GLMB filter for the purpose of tracking trajectories. The preliminary results of this work are reported in [40]. Particularly, in [40], the unknown detection probability is treated by the $p_{D}-$CPHD filter before boostraped into the GLMB filter with known clutter rate. The soundness and effectiveness of the proposed solution are demonstrated in Section 4 via a multiple marine ships tracking application.

The remainder of this work is presented as follows. The backgrounds on GLMB filtering will be given in Section 2. The proposed bootstrapping method will be introduced in Section 3 followed by numerical studies in Section 4. Some concluding marks in Section 5.

## 2. Background

Some fundamentals on multitarget state-space model, the CPHD filter, and GLMB filter will be summarized in this section. Following the convention in [11], single target states are denoted with lower-case letters (i.e., *x*) while upper-case letters denote multitarget states (i.e., *X*). The corresponding spaces are denoted by blackboard bold letters (X,L,Z,etc). The sequence of variable $X_{i},X_{i+1},...,X_{j}$ is abbreviated by $X_{i:j}$. In this work the inner product ∫f(x)g(x)dx is rewritten as 〈f,g〉. Given a set *S*, the finite subsets of *S* is written as F(S), and $1_{S}\left(\cdot \right)$ denotes the indicator function of *S*. For a finite set *X*, |X| represents its the number of elements, and the product $\prod_{x\in X}f\left(x\right)$ for some real-valued function *f* is denoted by the multitarget exponential $f^{X}$, with $f^{\emptyset }=1$. Further, the generalized Kronecker-delta function $\delta_{Y}$ whose arguments can be arbitrary sets, vectors, integers, etc., is defined as follows

(1) $$\delta_{Y}\left[X\right]=\left\{\begin{array}{cc} 1 & if\;X=Y\\ 0 & otherwise. \end{array}\right.$$

### 2.1. Multitarget States

As mentioned in Section 1, algorithms using non-labeled RFS cannot produce trajectories without using heuristic techniques [10]. The Labeled RFS framework, introduced in [11,41], is a principled approach to produce target tracks. Moreover, it is the only method that can produce trajectories from the filtering density [10]. In the labeled RFS frame work, a labeled target at time *k* is represented by a kinematic target state vector $x_{k}$ in state space X and its unique label $\ell_{k}$ in the (discrete) label space L, and hence x=(x,ℓ)∈X×L. This unique label is characterized by two parameters: time of target birth τ and the index of individual targets born at the same time ρ, i.e., $\ell_{k}=\left(\tau ,\rho \right)\in L$ [11]. Hence, formally, a trajectory of each target is a sequence of consecutive labeled states with the same label [11]. Note that the label space for all targets born up to time *k* is the disjoint union $L_{k}=L_{k-1}⊎B_{k}$ where $B_{k}$ is the label space for targets born at time k, and $L_{k-1}$ is the label space of the targets born prior to time *k*. To distinguish the unlabeled states from labeled ones, the normal and bold letters (e.g., x,X,x,X) are used, respectively. Suppose that at time *k*, there are *N* targets with corresponding states $x_{k,1},\ldots ,x_{k,N}$, then the multitarget state can be represented as follows:

(2) $$X_{k}=\left\{x_{k,1},\ldots ,x_{k,N}\right\}\in F\left(X\times L_{k}\right)$$

**Definition** **1.** *[11] Let L:X×L→L be the projection L(x;ℓ)=ℓ, and hence $L\left(X\right)=\left\{L\left(x\right):x\in X\right\}$ is the set of labels of X. A labeled RFS with space X and (discrete) label space L is an RFS on X×L such that each realization X has distinct labels, i.e., |L(X)|=|X|.*

Since each target in a multitarget state has a distict label, $\delta_{\left|X\right|}\left(|L\left(X\right)|\right)=1$, the distinct label indicator can be defined as follows [11]

(3) $$\Delta \left(X\right)≜\delta_{\left|X\right|}\left(|L\left(X\right)|\right).$$

### 2.2. Standard Multitarget Dynamic Model

Given a multitarget state $X_{k}$ at time *k*, each state $\left(x_{k},\ell_{k}\right)\in X_{k}$ can either exist with probability $P_{S,k+1|k}\left(x_{k}\right)$ and evolve to a new state $x_{k+1}$ at next time step k+1 with probability density $f_{k+1|k}\left(x_{k+1}|x_{k},\ell_{k}\right)\delta_{\ell_{k}}\left(\ell_{k+1}\right)$ or disappear with probability $1-P_{S,k+1|k}\left(x_{k}\right)$. Let $S_{k+1|k}\left(x\right)$ be the labeled Bernoulli RFS of the surviving target with state x from time *k* to time k+1 and $B_{k+1}$ be the labeled multi-Bernoulli RFS of the new-born targets at time k+1, then the multitarget state $X_{k+1}$ is the union of the surviving targets and the new-born ones,

(4) $$X_{k+1}=\underset{x_{k}\in X_{k}}{⋃}S_{k+1|k}\left(x_{k}\right)\cup B_{k+1},$$

Following the convention in [9], in this work, the set $B_{k+1}$ is distributed according to the labeled multi-Bernoulli (LMB) density. Furthermore, for simplicity, the subscript *k* for the current time is omitted, and the next time step k+1 is indicated by the subscript ${}^{\prime }{+}^{\prime }$.

Assuming that the appearance, disappearance, and movement of each target are independent of the others, the multitarget transition density (The Mahler’s Finite Set Statistics (FISST) notion of density is used in this paper for consistency with the probability density [42]) is [11,41]

(5) $$f\left(X_{+}|X\right)=f_{S}\left(X_{+}\cap \left(X\times L\right)|X\right)f_{B,+}\left(X_{+}-\left(X\times L\right)\right)$$

in which the distribution of new-born targets is given by

(6) $$\begin{array}{c} f_{B,+}\left(B_{+}\right)=\Delta \left(B_{+}\right){\left[1_{B_{+}}r_{B,+}\right]}^{L\left(B_{+}\right)}{\left[1-r_{B,+}\right]}^{B_{+}-L\left(B_{+}\right)}p_{B,+}^{B_{+}}, \end{array}$$

where $r_{B,+}\left(\ell \right)$ is the birth probability of new target with new-born label *ℓ*, and $p_{B,+}\left(\cdot ;\ell \right)$ is the distribution of its kinematic state [11]. The distribution of the survival targets is

(7) $$\begin{array}{cc} f_{S,+}\left(S|X\right)= & \Delta \left(S\right)\Delta \left(X\right)1_{L(X)}\left(L\left(S\right)\right){\left[\Upsilon (S;\cdot )\right]}^{X}\\ \Upsilon (S;x,\ell )= & \sum_{(x_{+},\ell_{+})\in S}\delta_{\ell }\left(\ell_{+}\right)P_{S}\left(x,\ell \right)f_{+}\left(x_{+}|x,\ell \right)+(1-1_{L\left(S\right)}\left(\ell \right)\left(1-P_{S}\left(x,\ell \right)\right). \end{array}$$

### 2.3. Standard Multitarget Observation Model

Assuming that there are *M* sensors, each state $\left(x,\ell \right)\in X$ can be either detected by sensor s,s=1,…M with probability of detection $P_{D}^{\left(s\right)}\left(x,\ell \right)$ and generate an observation $z^{\left(s\right)}\in Z^{\left(s\right)}$ with likelihood $g_{D}^{\left(s\right)}\left(z^{\left(s\right)}|x,\ell \right)$, or being miss detected with probability $1-P_{D}^{\left(s\right)}\left(x,\ell \right)$. The set of multitarget observations collected by the $s^{th}$-sensor at time *k* is $Z_{k}^{\left(s\right)}=\left\{z_{1}^{\left(s\right)},\ldots ,z_{M}^{\left(s\right)}\right\}\in F\left(Z\right)$, with Z being the observation space. Note that, the $s^{th}-$ sensor can also receive spurious measurements or false alarms at each time step. Let $D^{\left(s\right)}\left(x\right)$ be the set of measurements generated by target with state x at time *k*, the multitarget observation at the current time *k* is the superposition of all observations of detected targets modeled by multi-Bernoulli RFS, i.e., $D^{\left(s\right)}\left(X\right)={⋃}_{x\in X}D^{\left(s\right)}\left(x\right)$ and the clutter modeled by either Poisson or i.i.d. clutter RFS $C^{\left(s\right)}$.

(8) $$Z^{\left(s\right)}=D^{\left(s\right)}\left(X\right)\cup C^{\left(s\right)}$$

The likelihood function of a multitarget state X for sensor *s* is given as follows [9],

(9) $$g^{\left(s\right)}\left(Z^{\left(s\right)}|X\right)\;\propto \sum_{\theta^{\left(s\right)}\in \Theta^{\left(s\right)}}1_{\Theta^{\left(s\right)}\left(L\left(X\right)\right)}\left(\theta^{\left(s\right)}\right){\left[\Upsilon_{Z^{\left(s\right)}}^{\left(s,\theta^{\left(s\right)}\left(L\left(x\right)\right)\right)}\left(x\right)\right]}^{X}$$

where $\Theta^{\left(s\right)}$ is the set of positive association map $\theta^{\left(s\right)}$ at time k, $\theta^{\left(s\right)}:L\to \left\{0,1,\ldots ,\right|Z^{\left(s\right)}\left|\right\}$, such that $\left[\theta^{\left(s\right)}\left(i\right)=\theta^{\left(s\right)}\left(j\right)\right]\Rightarrow \left[i=j\right]$ (i.e., each observation in $Z^{\left(s\right)}$ is assigned to at most one target, then each target has a distinct label), $\Theta^{\left(s\right)}\left(J\right)$ is the subset of $\Theta^{\left(s\right)}$ with domain J, and

(10) $$\Upsilon_{Z^{\left(s\right)}}^{\left(s,j\right)}\left(x\right)=\left\{\begin{array}{cc} \frac{P_{D}^{\left(s\right)}\left(x\right)g^{\left(s\right)}\left(z_{j}^{\left(s\right)}|x\right)}{\kappa^{\left(s\right)}\left(z_{j}^{\left(s\right)}\right)}, & j=1:M^{\left(s\right)}\\ 1-P_{D}^{\left(s\right)}\left(x\right) & j=0. \end{array}\right.$$

Using the assumption that the sensors are conditionally independent (More concisely, the sensors do not interfere or influence each other while taking measurements or detections. The measurement noise, missed detections and clutter from each sensor in a multitarget scenario are, therefore, independent from the others), and let us define the following abbreviations

(11) $$\begin{array}{cc} Z & ≜(Z^{\left(1\right)},\cdots ,Z^{\left(M\right)}), \end{array}$$

(12) $$\begin{array}{cc} \Theta & ≜\Theta^{\left(1\right)}\times \cdots \times \Theta^{\left(M\right)}, \end{array}$$

(13) $$\begin{array}{cc} \Theta \left(J\right) & ≜\Theta^{\left(1\right)}\left(J\right)\times \cdots \times \Theta^{\left(M\right)}\left(J\right), \end{array}$$

(14) $$\begin{array}{cc} \theta & ≜(\theta^{\left(1\right)},\cdots ,\theta^{\left(M\right)}), \end{array}$$

(15) $$\begin{array}{cc} 1_{\Theta \left(I\right)}\left(\theta \right) & ≜\prod_{s=1}^{M}1_{\Theta^{\left(s\right)}\left(I\right)}\left(\theta^{\left(s\right)}\right), \end{array}$$

(16) $$\begin{array}{cc} \Upsilon_{Z}^{\left(j^{\left(1\right)},\cdots ,j^{\left(M\right)}\right)}\left(x,\ell \right) & ≜\prod_{s=1}^{M}\Upsilon_{Z^{\left(s\right)}}^{\left(s,j^{\left(s\right)}\right)}\left(x,\ell \right), \end{array}$$

then, the multi-sensor likelihood is written as

(17) $$\begin{array}{cc} g\left(Z|X\right)= & \prod_{s=1}^{M}g^{\left(s\right)}\left(Z^{\left(s\right)}|X\right)\\ \propto & \sum_{\theta \in \Theta }1_{\Theta \left(L\left(X\right)\right)}\left(\theta \right){\left[\Upsilon_{Z}^{\left(\theta \left(L\left(x\right)\right)\right)}\left(x\right)\right]}^{X}. \end{array}$$

Obviously, the form of the multi-sensor likelihood $g\left(Z|X\right)$ in (17) and that of it its single-sensor counterpart in (9) are identical.

### 2.4. Multitarget Bayesian Recursion

Let $\pi_{k-1}\left(\cdot |Z_{1:k-1}\right)$ denotes the multitarget density of the multitarget state at time k-1, where $Z_{1:k-1}=\left(Z_{1},\ldots ,Z_{k-1}\right)$ is the set of all observation history up to time k-1. For simplicity, we obmit the dependence on past measurements, i.e, we use $\pi_{k-1}\left(\cdot |Z_{k-1}\right)$ instead of $\pi_{k-1}\left(\cdot |Z_{1:k-1}\right)$. The multitarget Bayes filter use the Chapman-Kolmogorov equation to predict the multitarget state to time *k* given posterior at time k-1 as follows [3]

(18) $$\pi_{k|k-1}\left(X_{k}|Z_{k-1}\right)=\int f_{k|k-1}\left(X_{k}|X\right)\pi_{k-1}\left(X|Z_{k-1}\right)dX,$$

where $f_{k|k-1}\left(X_{k}|X\right)$ is defined as the multitarget transition kernel from time k-1 to time *k*, and the integral in Equation (18) is the set integral defined for any function $f:F\left(X\times L\right)\to R$,

(19) $$\int f\left(X\right)\delta X=\sum_{i=0}^{\infty }\frac{1}{i!}\int f\left(\left\{x_{1},\ldots ,x_{i}\right\}\right)d\left(x_{1},\ldots ,x_{i}\right).$$

The multitarget state $X_{k}$ is partially observed at time *k*, and the RFS $Z_{k}$ is modeled by the multitarget likelihood function $g_{k}\left(Z_{k}|X_{k}\right)$, thus the multitarget posterior at this time is given by Bayes rule:

(20) $$\pi_{k}\left(X_{k}|Z_{k}\right)=\frac{g_{k}\left(Z_{k}|X_{k}\right)\pi_{k|k-1}\left(X_{k}|Z_{k-1}\right)}{\int g_{k}\left(Z_{k}|X\right)\pi_{k|k-1}\left(X|Z_{k-1}\right)dX}.$$

## 3. GLMB Recursion with Bootstrapping Method

In this section, the generalized labeled multi-Bernoulli (GLMB) filter with its recursion is summarized. The proposed method for estimating unknown backgrounds before bootstrapping them into this filter is also introduced.

### 3.1. GLMB Filter

**Definition** **2.** *A GLMB density is a labeled multitarget density given as follows [11]*

(21) $$\pi \left(X\right)=\Delta \left(X\right)\sum \sum_{\varrho \in \Xi ,J\subseteq L}\omega^{\left(J,\varrho \right)}\delta_{J}\left[L\left(X\right)\right]{\left[p^{\left(\varrho \right)}\right]}^{X},$$

*where the discrete space *Ξ* is the space of association map histories $\Theta_{0:k}≜\Theta_{0}\times \ldots \times \Theta_{k}$, each $\varrho =\left(\theta_{1:k}\right)\in \Xi$ represents a history of the (multisensor) positive 1-1 map, the weight $\omega^{\left(J,\varrho \right)}$ and multitarget exponential ${\left[p^{\left(\varrho \right)}\right]}^{X}$ satisfy*

(22) $$\begin{array}{cc} \sum_{\varrho \in \Xi }\sum_{J\in L}\omega^{\left(J,\varrho \right)}\delta_{J}\left[L\left(X\right)\right] & =1, \end{array}$$

(23) $$\begin{array}{cc} \int p^{\left(\varrho \right)}\left(x,\ell \right)dx & =1. \end{array}$$

Noting that, in Equation (21), while $\omega^{\left(J,\varrho \right)}\left(L\left(X\right)\right)$ is a function of only the labels of the multitarget state X, whereas ${\left[p^{\left(\varrho \right)}\right]}^{X}$ depends on entire set X.

The cardinality distribution $Pr\left(|X|=n\right)$, existence probability $r\left(\ell \right)$ and probability density $p\left(x,\ell \right)$ of a track ℓ∈L are given as follows [11]:

(24) $$\begin{array}{cc} Pr\left(|X|=n\right) & =\sum_{\varrho \in \Xi }\sum_{J\in L}\delta_{n}\left[|J|\right]\omega^{\left(J,\varrho \right)} \end{array}$$

(25) $$\begin{array}{cc} r\left(\ell \right) & =\sum_{\varrho \in \Xi }\sum_{J\in L}1_{J}\left(\ell \right)\omega^{\left(J,\varrho \right)} \end{array}$$

(26) $$\begin{array}{cc} p\left(x,\ell \right) & =\frac{1}{r\left(\ell \right)}\sum_{\varrho \in \Xi }\sum_{J\in L}1_{J}\left(\ell \right)\omega^{\left(J,\varrho \right)}p^{\left(\varrho \right)}\left(x,\ell \right) \end{array}$$

#### 3.1.1. The GLMB Recursion

Since the GLMB filter is an exact closed-form multitarget Bayes filter under the standard multitarget dynamic and observation models [12], and the form of the likelihood function in a single sensor and multisensor cases are identical, the GLMB filter can be implemented via two separate steps (update and prediction) or the combined step (joint-predict-update process). In this work, for the convenience of proposed method, the two step GLMB recursion will be presented.

##### *a. GLMB update* 

Given the standard multitarget observation likelihood function (9), the posterior multitarget density is calculated as follows [11]

(27) $$\begin{array}{c} \pi^{\left(s\right)}\left(X|Z^{\left(s\right)}\right)=\Delta \left(X\right)\sum_{(J,\varrho )\in F(L)\times \Xi }\sum_{\theta^{\left(s\right)}\in \Theta }\omega_{\left(Z^{\left(s\right)}\right)}^{\left(s,J,\varrho ,\theta^{\left(s\right)}\right)}\left(L\left(X\right)\right){\left[p^{\left(s,\varrho ,\theta^{\left(s\right)}\right)}\left(\cdot |Z^{\left(s\right)}\right)\right]}^{X}, \end{array}$$

where

(28) $$\begin{array}{cc} \omega_{Z^{\left(s\right)}}^{\left(s,J,\varrho ,\theta^{\left(s\right)}\right)}\left(L\right) & =\frac{\Gamma_{Z^{\left(s\right)}}^{\left(s,J,\varrho ,\theta^{\left(s\right)}\right)}}{\sum_{(J,\varrho )\in F(L)\times \Xi }\sum_{\theta^{\left(s\right)}\in \Theta }\Gamma_{Z^{\left(s\right)}}^{\left(s,J,\varrho ,\theta^{\left(s\right)}\right)}} \end{array}$$

(29) $$\begin{array}{cc} \Gamma_{Z^{\left(s\right)}}^{\left(s,J,\varrho ,\theta^{\left(s\right)}\right)} & =\omega_{Z^{\left(s\right)}}^{\left(s,J,\varrho \right)}\left(L\right){\left[{\bar{p}}_{Z^{\left(s\right)}}^{\left(s,\varrho ,\theta^{\left(s\right)}\right)}\right]}^{J} \end{array}$$

(30) $$\begin{array}{cc} p^{\left(s,\varrho ,\theta^{\left(s\right)}\right)}\left(x,\ell |Z^{\left(s\right)}\right) & =\frac{p^{\left(s,\varrho \right)}\left(x,\ell \right)\Upsilon_{Z^{\left(s\right)}}^{\left(s\right)}\left(x,\ell ;\theta^{\left(s\right)}\right)}{{\bar{p}}_{Z^{\left(s\right)}}^{\left(s,\varrho ,\theta^{\left(s\right)}\right)}\left(\ell \right)} \end{array}$$

(31) $$\begin{array}{cc} {\bar{p}}_{Z^{\left(s\right)}}^{\left(s,\varrho ,\theta^{\left(s\right)}\right)}\left(\ell \right) & =\langle p^{\left(s,\varrho \right)}\left(\cdot ,\ell \right),\Upsilon_{Z^{\left(s\right)}}^{\left(s\right)}\left(\cdot ,\ell ;\theta^{\left(s\right)}\right)\rangle \end{array}$$

and $\Upsilon_{Z^{\left(s\right)}}^{\left(s\right)}\left(x,\ell ;\theta^{\left(s\right)}\right)$ is given in (10).

##### *b. Prediction* 

Given the posterior multitarget density at current time is a GLMB filtering density with the form of (21), the predicted multitarget density at next time step is calculated under the standard multitarget dynamic model (4) as follows [11]:

(32) $$\pi_{+}^{\left(s\right)}\left(X_{+}\right)=\Delta \left(X_{+}\right)\sum_{\left(J_{+},\varrho \right)\in F\left(L_{+}\right)\times \Xi }\omega_{+}^{\left(s,J_{+},\varrho \right)}\left(L\left(X_{+}\right)\right){\left[p_{+}^{\left(s,\varrho \right)}\right]}^{X_{+}}$$

where

(33) $$\begin{array}{cc} \omega_{+}^{\left(s,J_{+},\varrho \right)}\left(L\right)= & \omega_{B}^{\left(s\right)}\left(J_{+}\cap B\right)\omega_{S}^{\left(s,\varrho \right)}\left(J_{+}\cap L\right), \end{array}$$

(34) $$\begin{array}{cc} p_{+}^{\left(s,\varrho \right)}\left(x,\ell \right)= & 1_{L}\left(\ell \right)p_{S}^{\left(s,\varrho \right)}\left(x,\ell \right)+\left(1-1_{L}\left(\ell \right)\right)p_{B}^{\left(s\right)}\left(x,\ell \right) \end{array}$$

(35) $$\begin{array}{cc} p_{S}^{\left(s,\varrho \right)}\left(x,\ell \right)= & \frac{\langle P_{S}^{\left(s\right)}\left(\cdot ,\ell \right)f\left(x|\cdot ,\ell \right),p^{\left(s,\varrho \right)}\left(\cdot ,\ell \right)\rangle }{{\bar{p}}_{S}^{\left(s,\varrho \right)}\left(\ell \right)}, \end{array}$$

(36) $$\begin{array}{cc} {\bar{p}}_{S}^{\left(s,\varrho \right)}\left(\ell \right)= & \int \langle P_{S}^{\left(s\right)}\left(\cdot ,\ell \right)f\left(x|\cdot ,\ell \right),p^{\left(s,\varrho \right)}\left(\cdot ,\ell \right)\rangle dx \end{array}$$

(37) $$\begin{array}{cc} \omega_{S}^{\left(s,\varrho \right)}\left(L\right)= & {\left[{\bar{p}}_{S}^{\left(s,\varrho \right)}\right]}^{L}\sum_{J\subseteq L}1_{J}\left(L\right){\left[Q_{S}^{\left(s,\varrho \right)}\left(\ell \right)\right]}^{J-L}\omega^{\left(s,J,\varrho \right)} \end{array}$$

(38) $$\begin{array}{cc} Q_{S}^{\left(s,\varrho \right)}\left(\ell \right)= & \langle 1-P_{S}^{\left(s\right)}\left(\cdot ,\ell \right),p^{\left(s,\varrho \right)}\left(\cdot ,\ell \right)\rangle . \end{array}$$

### 3.2. Adaptive to Unknown Backgrounds

In practice, the both the clutter rate and detection profile are unknown and unpredictably vary with time. Prior knowledge of background models, therefore, are typically unavailable. Mismatch in background models results in degradation of tracker performance [4]. In this section, based on the suite of methods for tackling the unknown clutter rate and detection probability introduced in [4], the bootstrapping method will be proposed.

A technique that accommodates the jointly unknown clutter rate λ and the unknown probability of detection $p_{D}$ has been introduced in [29]. This technique considers clutter as an RFS of "generator targets” or “false targets”, and incorporates the non-homogeneous and unknown detection probability into each target state. Each real target state x∈X is corresponded to an augmented state $x_{a}=\left(x,a\right),$ in which $a\in X^{d}=\left[0,1\right]$ is the variable on the probability detecting *x*. The augmented multitarget state now can be described as follows

(39) $$X_{a}=\left(x_{a,1},\ldots ,x_{a,n}\right)=\left\{\left(x_{1},a_{1}\right),\ldots ,\left(x_{n},a_{n}\right)\right\}$$

Similarly, the augmented generator target state is $x_{c}=\left[\bar{x},a_{c}\right]$ with $\bar{x}\in X^{c}$ be the generator target state, and $a_{c}\in X^{d}=\left[0,1\right]$. The augmented generator multitarget state is

(40) $$X_{c}=\left(x_{c,1},\ldots ,x_{c,m}\right)=\left\{\left({\bar{x}}_{1},a_{c1}\right),\ldots ,\left({\bar{x}}_{n},a_{cm}\right)\right\}$$

Then the probability of detection is replaced by *a* and $a_{c}$, respectively.

(41) $$\begin{array}{cc} p_{D,a}^{\left(s\right)}\left(x_{a}\right) & =p_{D,a}^{\left(s\right)}\left(x,a\right)≜a \end{array}$$

(42) $$\begin{array}{cc} p_{D,c}^{\left(s\right)}\left(x_{c}\right) & =p_{D,c}^{\left(s\right)}\left(\bar{x},a_{c}\right)≜a_{c} \end{array}$$

Assuming that the false and true targets are statistically independent, then each of the augmented generator targets can be modeled for their characteristics as appearances, disappearances, and transitions, together with likelihood, detection and missed detection. The multitarget state is then a combination of (augmented) actual targets and clutter generators. Meaning that, the augmented hybrid space $X^{h}$ involving the multitarget state can be defined as follows [29]

(43) $$X^{\left(h\right)}=\left(X\times X^{\left(d\right)}\right)⊎\left(X^{\left(c\right)}\times X^{\left(d\right)}\right)≜X_{a}^{\left(d\right)}⊎X_{c}^{\left(d\right)}$$

where “⊎” denotes the disjoint union, and “×” denotes the Cartesian product.

The multitarget state (4) and multitarget observation (8)) at time *k* now become the hybrid ones:

(44) $$\begin{array}{cc} X_{h} & =X_{a}⊎X_{c} \end{array}$$

(45) $$\begin{array}{cc} Z_{h} & =Z_{a}⊎Z_{c} \end{array}$$

with $Z_{a}$ and $Z_{c}$ be the augmented multitarget and augmented generator observations, respectively. The integral of a function $f^{\left(h\right)}:X^{\left(h\right)}\to R$ is given by [29]

(46) $$\int_{X^{\left(h\right)}}f^{\left(h\right)}\left(X_{h}\right)dx_{h}=\int_{X_{a}^{\left(d\right)}}f_{a}^{\left(d\right)}\left(X_{a}\right)\delta X_{a}+\int_{X_{a}^{c}}f_{a}^{\left(c\right)}\left(X_{c}\right)\delta X_{c}$$

Here, the set integral (19) has been applied to both augmented multitarget state and augmented generator multitarget state terms, i.e, [4]

(47) $$\begin{array}{cc} \int f_{a}^{\left(d\right)}\left(X_{a}\right)\delta X_{a} & =\sum_{n\geq 0}\frac{1}{n!}f_{a}\left(\left\{x_{a,1},\ldots ,x_{a,n}\right\}\right)dx_{a,1},\ldots ,dx_{a,n} \end{array}$$

(48) $$\begin{array}{cc} \int f_{c}^{\left(d\right)}\left(X_{c}\right)\delta X_{c} & =\sum_{m\geq 0}\frac{1}{m!}f_{a}^{c}\left(\left\{x_{c,1},\ldots ,x_{c,n}\right\}\right)dx_{c,1},\ldots ,dx_{c,m} \end{array}$$

Noting that the the measurement likelihood is kept unchanged

(49) $$\begin{array}{cc} g\left(x_{a}\right) & =g_{a}^{\left(s\right)}\left(x,a\right)≜g^{\left(s\right)}\left(x\right) \end{array}$$

(50) $$\begin{array}{cc} g\left(x_{c}\right) & =g_{c}^{\left(s\right)}\left(\bar{x},a_{c}\right)≜g^{\left(s\right)}\left(\bar{x}\right) \end{array}$$

**Figure 1:**
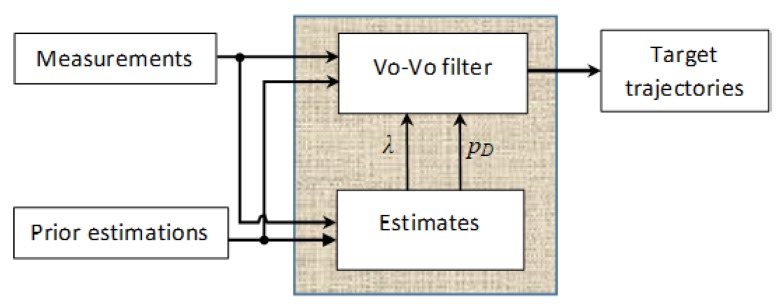
The proposed structure of the *B*-GLMB filter.

While the method proposed in [29] results in good estimates of targets, it do not produce the trajectories of the targets. Moreover, although this method is a closed-form solution of the CPHD recursion with jointly unknown clutter rate and detection profile, it is proposed for single-sensor multiple targets estimation solely. In this paper, we propose a method of using the technique introduced in [29] to estimate the mentioned unknown parameters then bootstrapping them into the GLMB filter for tracking on-the-fly. The structure of the proposed method is given in Figure 1.

### 3.3. Implementation

Since after each filtering iteration, the number of components in the GLMB density grows at an exponential rate, the low weight terms should be truncated for tractability. In this work, we use Murty’s ranked assignment algorithm to sample a given number of hypotheses of the multitarget density with the highest probability to be the correct ones. Then these components are propagated through the filtering recursion only. Although the use of Murty’s algorithm leads to a cubic complexity in the product of the number of Doppler measurements, its implementation is reasonable because there are maximum 10 targets in this work.

## 4. Numerical Study

The advantages of multi-static Doppler radar such as lightweight, wide range of surveillance with high accuracy, and low power consumption lead to its broad applications in both civilian and military applications [43,44,45]. However, the number of the sensors in conjunction with the non-linear nature and and low observability of the Doppler type measurement leads to many numerical difficulties [44,45]. The use of multistatic Doppler-only measurements in a scenario of 10 receivers and one cooperative transmitter has been proposed in [46] and its extended version [47] for joint detection and tracking of one target.

This numerical study based on the model mentioned in [40] with 10 marine ships. Each ship at time *k* is represented by a 5-D state vector $x_{k}$ in the surveillance of interest $x_{k}={\left[p_{k}^{T},\nu_{k}^{T},\alpha_{k}\right]}^{T},$ where $p_{k}={\left[\mu_{k},\lambda_{k}\right]}^{T}$ and $\nu_{k}={\left[{\dot{\mu }}_{k},{\dot{\lambda }}_{k}\right]}^{T}$ denote the position and velocity in the longitude and latitude, respectively; $\alpha_{k}$ is the course of the target, and *T* denotes the transpose operation. The target dynamic model can be given as follows:

(51) $$x_{k}=F_{k|k-1}\left(x_{k-1}\right)+Gn_{k}$$

where

(52) $$\begin{array}{cc} F\left(x_{k-1}\right) & =\left[\begin{array}{ccccc} 1 & \frac{\sin(\alpha t)}{\alpha } & 0 & \frac{\left(\cos(\alpha t)-1\right)}{\alpha } & 0\\ 0 & \cos(\alpha t) & 0 & -\sin(\alpha t) & 0\\ 0 & -\frac{\left(\cos(\alpha t)-1\right)}{\alpha } & 1 & -\frac{\sin(\alpha t)}{\alpha } & 0\\ 0 & \sin(\alpha t) & 0 & \cos(\alpha t) & 0\\ 0 & 0 & 0 & 0 & 1 \end{array}\right]x_{k-1};\;G=\left[\begin{array}{ccc} \frac{t^{2}}{2} & 0 & 0\\ t & 0 & 0\\ 0 & \frac{t^{2}}{2} & 0\\ 0 & t & 0\\ 0 & 0 & t \end{array}\right], \end{array}$$

and *t* is sample period, $n_{k}$ is a Gaussian noise vector of velocity and course noise components with zero-mean. Note that latitudinal and longitudinal measurements are in degrees ${(}^{\circ })$, the distance, speed and time are given in nautical miles (M), knots (kn), and hours (h), respectively.

**Remark** **1.** *Equation *(52)* is resulted from the assumption that the surveillance region is not very far from the Equator.*

The new births are assumed to be distributed with labeled multi-Bernoulli RFS distributions of parameters $f_{B}\left(x\right)={\left\{r_{B}^{\left(i\right)},p_{B}^{\left(i\right)}\right\}}_{i=1}^{4}$ where $r_{B}^{\left(i\right)}$ is the i^th^ common existence probability, and $p_{B}^{\left(i\right)}\left(x\right)=N\left(x,{\hat{x}}_{B}^{\left(i\right)},P_{B}\right)$ with

$$\begin{array}{cc} {\hat{x}}_{B}^{\left(1\right)} & ={\left[15.6^{\circ }N,0,113^{\circ }E,0,0\right]}^{T};\\ {\hat{x}}_{B}^{\left(2\right)} & ={\left[13.2^{\circ }N,0,107.5^{\circ }E,0,0\right]}^{T}\\ {\hat{x}}_{B}^{\left(3\right)} & ={\left[18.2^{\circ }N,0,110.7{}^{\circ }E,0,0\right]}^{T};\\ {\hat{x}}_{B}^{\left(4\right)} & ={\left[22.3^{\circ }N,0,118.8^{\circ }E,0,0\right]}^{T};\\ P_{B} & =diag\left(\left[2^{\prime }N,30\left(kn\right),2^{\prime }E,30\left(kn\right),6\pi /180\left(rads^{-1}\right)\right]\right) \end{array}$$

Table 1 lists out the initial state of ten targets with random time of appearance and disappearance, and the average course is $\bar{\alpha }=2\pi /180\left(rad/s\right).$

The parameters of the dynamic model are given in Table 2.

**Figure 2:**
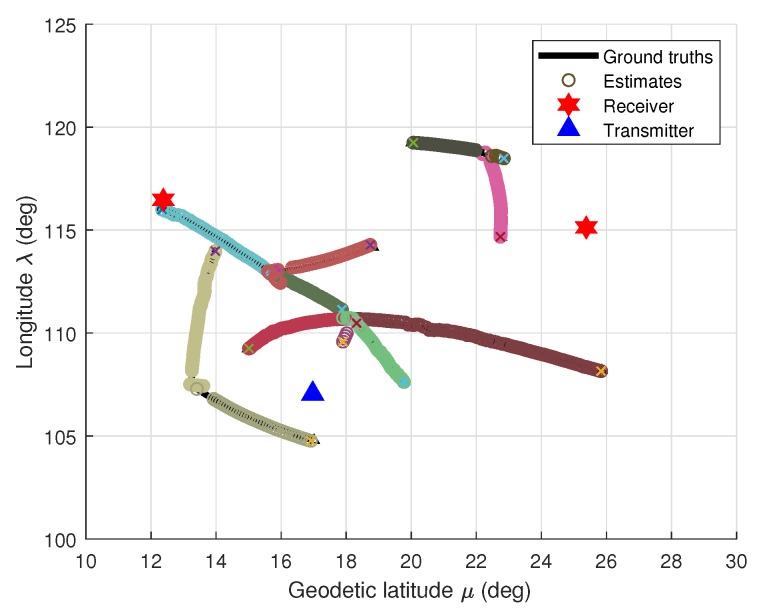
Configuration of multitarget tracking using MRS.

Consider the configuration of multiple Doppler sensors system including two spatially distributed receivers and one cooperative transmitter located as in Figure 2. Based on Doppler effect, this system can measure the speed of a target at a distance by calculating the altered frequency of the returned signals which originate from the emitting pulses of radio signals and being reflected to radar after reaching target [48].

The observation of a target state $x_{k}$ at the $s^{th}$ receiver using Doppler measurement is given by

(53) $$z_{k}^{\left(s\right)}=-\nu_{k}^{T}\left(\frac{p_{k}-p_{r}^{\left(s\right)}}{∥p_{k}-p_{r}^{\left(s\right)}∥}+\frac{p_{k}-p_{t}}{∥p_{k}-p_{t}∥}\right)\frac{f_{t}}{c}+w_{k},$$

in which $p_{k}$ and $\nu_{k}$ have been defined above the Equation (51), $p_{t}={\left[\mu_{t},\lambda_{t}\right]}^{T}$ is the position of the transmitter, and $p_{r}^{\left(s\right)}={\left[\mu_{r}^{\left(s\right)},\lambda_{r}^{\left(s\right)}\right]}^{T}$ is the location of the $s^{th}-$receiver; $w_{k}$ is zero-mean Gaussian noise, $w_{k}∼N\left(0,Q_{k}\right)$, with covariance $Q_{k}=diag\left([1Hz^{2}]\right)$; and $f_{t}$ is the signal frequency emitted from the transmitter, and *c* is the light speed.

Since the targets are dynamic in different directions, the value of observation $z_{k}^{\left(s\right)}$ in (53) can be negative or positive in the known interval $\left[-f_{0},+f_{0}\right]$ of the Doppler sensor. In this work, the measurement space for two receivers have the same measurement space of $\left[-200Hz,200Hz\right].$ The parameters of the observation model are given in Table 3. It can be seen that, not only the state equation but also the measurement one are highly nonlinear.

**Figure 3:**
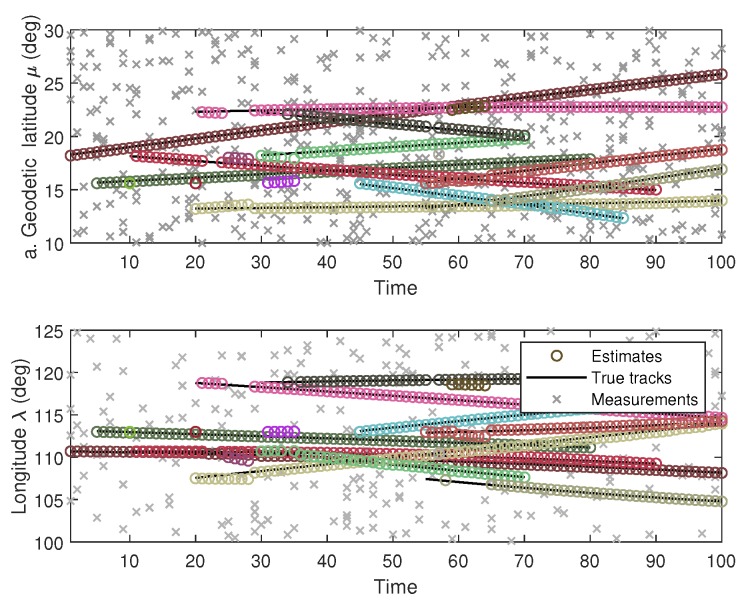
Tracking in longitudinal-latitudinal coordinates.

By using the proposed B-GLMB filter, the configuration of multiple marine ships tracking using multiple Doppler radars with ground truths and their tracking results are illustrated in Figure 2. For better visualization of multiple targets, each target is assigned to a distinct color. The results of longitudinal-latitudinal co-ordinate target trajectories are demonstrated in Figure 3.

**Figure 4:**
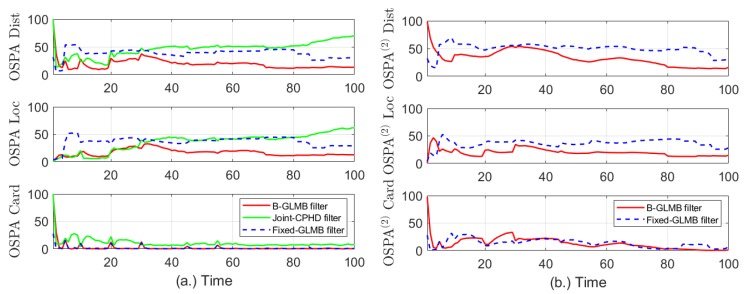
Evaluation of tracking errors using (**a**) OSPA, and (**b**) OSPA ^(2)^.

For evaluating the effectiveness of the proposed method comparing to the fixed-GLMB filter and the Joint-CPHD, 100 Monte - Carlo run has been used, and the distance, location and cardinality errors are calculated via Optimal Sub-Pattern Assignment, OSPA, [49] and shown in Figure 4a. By using this metric, the distance between the set of true multitarget states and that of estimated target states is calculated at each time step. For measuring the error between two set of tracks, the use of OSPA is insufficient, and OSPA ${}^{\left(2\right)}$ is needed. The OSPA ${}^{\left(2\right)}$ errors [50] of the B-GLMB and fixed-GLMB filters are compared and plotted against time in Figure 4b, respectively.

For the B-GLMB filter and joint-CPHD filter, the clutter rate fluctuates in the range of $\lambda_{c}=\left[28,70\right]$, and the detection probability changes from 0.75 to 0.98, i.e., $p_{D}=\left[0.75,0.98\right]$. The fixed-GLMB filter is used with fixed $p_{D}$ of 0.75 and 0.98 and fixed λ of 28 and 70, respectively. The window length used in OSPA ${}^{\left(2\right)}$ to obtain the differences between the true and estimate sets of trajectories in Figure 4b is set at $w_{l}=10$. Both the OSPA and OSPA ${}^{\left(2\right)}$ are used with cut-off parameter $c_{0}=0$ and p=1.

**Figure 5:**
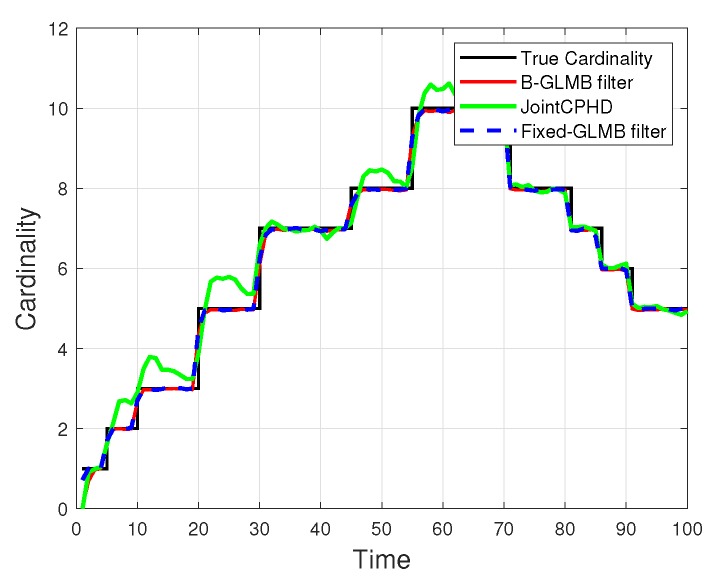
Cardinality tracking results.

Obviously from Figure 4a, the errors in distance and location between the set of true targets and the estimated ones using B-GLMB filter is the smallest values comparing to those of the fixed-GLMB and joint-CPHD filters. In addition, the errors in cardinality statistics using fixed-GLMB and B-GLMB are almost identical and better than error measured by joint-CPHD filter. The results of measuring errors between the set of true target tracks and that of the estimated tracks are given in Figure 4b. Once again, the effectiveness of the proposed method in reducing the errors in distances and locations of the target tracks is validated. The cardinality statistics for the B-GLMB filter, fixed-GLMB filter and joint-CPHD over 100 Monte Carlo run are given in Figure 5.

## 5. Conclusions

This paper presented an efficient solution to the problem of tracking an unknown and time-varying number of marine ships from multiple sensors with unknown clutter rate and probability of detection. Particularly, these two unknown parameters are parallel estimated based on the λ-CPHD and the $p_{D}-$CPHD filters, then bootstrapped into the cutting-edge GLMB filter. By using the bootstrapping method, the proposed filter utilizes the advantages of the two former estimators in accommodating the unknown backgrounds and reduces the computational cost from tracking algorithm of the latter filter. The effectiveness and correctness of the proposed method are demonstrated in Section 4. From our best knowledge, this is the first principled online algorithm for tracking marine ships via multiple sensors with unknown backgrounds in Doppler measurements. For future work, one of the forcuses would be investigating the combination of multi-scan GLMB [28] and multisensor GLMB [9] filters for multisensor multitarget tracking.

## Figures and Tables

**Table 1:** Target initial states.

Target	$\mu_{k}$	$\lambda_{k}$	$\dot{\mu_{k}}$	${\dot{\lambda }}_{k}$	$\alpha_{k}$ (rad/s)	Time of Birth (h)	Time of Beath (h)
1	$18^{\circ }12^{\prime }15^{\prime \prime }$	$110^{\circ }42^{\prime }06^{\prime \prime }$	32	-5	$-5\bar{\alpha }/8$	1	100
2	$15^{\circ }37^{\prime }52^{\prime \prime }$	$113^{\circ }57^{\prime }14^{\prime \prime }$	13	-9	$-\bar{\alpha }/2$	5	80
3	$18^{\circ }11^{\prime }40^{\prime \prime }$	$110^{\circ }41^{\prime }43^{\prime \prime }$	-18	0	$2\bar{\alpha }$	10	90
4	$13^{\circ }13^{\prime }52^{\prime \prime }$	$107^{\circ }29^{\prime }31^{\prime \prime }$	2	32	$-\bar{\alpha }/4$	20	100
5	$22^{\circ }17^{\prime }11^{\prime \prime }$	$118^{\circ }49^{\prime }24^{\prime \prime }$	6	-20	$-5\bar{\alpha }/6$	20	100
6	$22^{\circ }17^{\prime }58^{\prime \prime }$	$118^{\circ }48^{\prime }05^{\prime \prime }$	-22	6	$3\bar{\alpha }/4$	30	70
7	$18^{\circ }12^{\prime }15^{\prime \prime }$	$110^{\circ }42^{\prime }06^{\prime \prime }$	15	-30	$\bar{\alpha }/8$	30	70
8	$15^{\circ }35^{\prime }57^{\prime \prime }$	$113^{\circ }01^{\prime }06^{\prime \prime }$	-30	32	$3\bar{\alpha }/5$	45	85
9	$13^{\circ }11^{\prime }44^{\prime \prime }$	$107^{\circ }30^{\prime }19^{\prime \prime }$	28	-30	$5\bar{\alpha }/3$	55	100
10	$15^{\circ }36^{\prime }04^{\prime \prime }$	$112^{\circ }53^{\prime }30^{\prime \prime }$	30	5	$7\bar{\alpha }/4$	55	100

**Table 2:** Parameters of the Dynamic model.

Parameter	Symbol	Value
Sample period	*t*	0.15 (h)
Std. of speed noise	$\sigma_{\nu }$	2 (kn)
Std. of course noise	$\sigma_{\alpha }$	π/180 (rads ^−1^)
Common existence prob.	$r_{B}^{\left(1,2\right)},r_{B}^{\left(3,4\right)}$	(0.04; 0.02)
Survival prob.	$P_{S}$	0.95
Number of targets	*N*	10

**Table 3:** Parameters of observation model.

Name	Symbol	Value
Transmitter	$p_{t}$	$\left[16^{\circ }58^{\prime }16^{\prime \prime }N,107.02^{\prime }48^{\prime \prime }E\right]$
Receiver 1	$p_{r}^{\left(1\right)}$	$\left[12^{\circ }22^{\prime }43^{\prime \prime }N,116^{\circ }28^{\prime }25^{\prime }E\right]$
Receiver 2	$p_{r}^{\left(2\right)}$	$\left[25^{\circ }22^{\prime }47^{\prime \prime }N,115^{\circ }07^{\prime }19^{\prime \prime }E\right]$
Transmit freq.	$f_{t}$	300(Mhz)
Light speed	*c*	$3\times 10^{8}\;\left(m/s\right)$
Detection prob.	$p_{D}$	$\left[0.75;0.98\right]$
Clutter rate range	$\lambda_{c}$	$\left[28;60\right]$
Surveil. area	$S_{r}$	$\left[10^{\circ }-30^{\circ }N,100^{\circ }-125^{\circ }E\right]$
